# Guidance for the Management of Rectal Cancer: An Umbrella Review of Existing Guidelines Regarding Surgical Anatomy, Adjuvant Therapy, Follow-Up and Surveillance, Specific Considerations, Documentation, and Palliative Care

**DOI:** 10.7759/cureus.83345

**Published:** 2025-05-02

**Authors:** Ionut Negoi

**Affiliations:** 1 Department of General Surgery, Carol Davila University of Medicine and Pharmacy, Bucharest, ROU; 2 Department of General Surgery, Clinical Emergency Hospital of Bucharest, Bucharest, ROU

**Keywords:** adjuvant therapy, follow-up and surveillance, guidance, rectal cancer, surgical anatomy of the rectum, total mesorectal excision

## Abstract

This umbrella review systematically addresses current practices in the management of rectal cancer, covering pertinent terminology, precise surgical anatomy, adjuvant therapeutic regimens, postoperative follow-up protocols, clinical considerations for specific patient scenarios, standards for operative and pathological documentation, and effective palliative care approaches. Emphasis is placed on the essential function of multidisciplinary collaboration and the utilization of uniform terminology to facilitate precise communication and enhance clinical outcomes.

The surgical anatomy of the rectum is delineated, specifying the upper anatomical boundary, the surgical anal canal, and the segmentation of the rectum into lower, middle, and upper thirds. Additionally, the review accentuates the clinical relevance of anatomical structures such as the mesorectal fascia, anal sphincter complex, and lateral pelvic lymph nodes, highlighting their critical roles in precise tumor staging and surgical decision-making.

Evidence-based recommendations for adjuvant therapeutic approaches are clearly presented, stratified according to tumor stage and established risk factors. Comprehensive guidelines for postoperative surveillance incorporate regular clinical assessments, monitoring of carcinoembryonic antigen (CEA) levels, utilization of appropriate imaging techniques, scheduled colonoscopies, and systematic evaluation of patient-reported quality-of-life outcomes.

The review specifically addresses complex clinical circumstances, including therapeutic strategies for metastatic (stage IV) rectal cancer, management protocols for acute complications such as hemorrhage, bowel obstruction, and perforation, as well as the importance of rigorous preoperative patient optimization and prehabilitation strategies. Additionally, it delineates detailed standards for documentation within operative and pathology reports and elaborates on evidence-based practices in palliative care management for advanced rectal cancer cases.

Lastly, the review underscores the necessity of implementing national databases for comprehensive data collection, fostering specialized training programs for colorectal surgeons, establishing accredited centers of excellence, and conducting systematic audits. These initiatives are crucial to maintaining and enhancing the quality of clinical care and continuously improving patient outcomes.

## Introduction and background

Standardized evidence-based guidelines developed by multidisciplinary teams (MDT) have become essential for the effective management of rectal cancer. Despite efforts towards standardization, considerable variation still exists among international guidelines regarding definitions, treatment modalities, and surveillance recommendations for rectal cancer management [1]. These variations emphasize the necessity for an updated review that can reveal both common recommendations and differences, thereby encouraging a harmonized approach to patient care [2]. 

The objective of this umbrella review was to align the recommendations outlined in international guidelines concerning rectal cancer. It seeks to identify supporting evidence regarding standard terminology, surgical anatomy, adjuvant therapy, follow-up strategies, management of specific clinical scenarios, documentation standards, and palliative care with the aim of enhancing consistency in clinical practice.

## Review

Methods

We performed an umbrella review, with a systematic search of the PubMed/MEDLINE (see Table 1) and Web of Science (see Table 2) databases for published guidelines. The search strategy used in PubMed/MEDLINE was as follows: (((rectal cancer[Title/Abstract]) OR (rectal adenocarcinoma[Title/Abstract])) OR (rectal neoplasm[Title/Abstract])) AND ((((guideline[Title/Abstract])) OR (guidance[Title/Abstract]))).

**Table 1:** Search strategy used in PubMed/MEDLINE.

Search number	Query	Search details	Results
3	(((rectal cancer[Title/Abstract]) OR (rectal adenocarcinoma[Title/Abstract])) OR (rectal neoplasm[Title/Abstract])) AND ((((guideline[Title/Abstract])) OR (guidance[Title/Abstract])))	("rectal cancer"[Title/Abstract] OR "rectal adenocarcinoma"[Title/Abstract] OR "rectal neoplasm"[Title/Abstract]) AND ("guideline"[Title/Abstract] OR "guidance"[Title/Abstract])	441
2	(((guideline[Title/Abstract])) OR (guidance[Title/Abstract]))	"guideline"[Title/Abstract] OR "guidance"[Title/Abstract]	284,006
1	((rectal cancer[Title/Abstract]) OR (rectal adenocarcinoma[Title/Abstract])) OR (rectal neoplasm[Title/Abstract])	"rectal cancer"[Title/Abstract] OR "rectal adenocarcinoma"[Title/Abstract] OR "rectal neoplasm"[Title/Abstract]	34,018

**Table 2:** Search strategy used in the Web of Science Core Collection.

#	Search query	Database	Results
1	((ALL=(rectal cancer)) OR ALL=(rectal adenocarcinoma)) OR ALL=(rectal neoplasm)	Web of Science Core Collection	74,223
2	(ALL=(guideline)) OR ALL=(guidance)	Web of Science Core Collection	1,438,866
3	#1 AND #2	Web of Science Core Collection	4,682
4	(TI=(guideline)) OR TI=(guidance)	Web of Science Core Collection	178,857
5	((TI=(rectal cancer)) OR TI=(rectal adenocarcinoma)) OR TI=(rectal neoplasm)	Web of Science Core Collection	31,377
6	#4 AND #5	Web of Science Core Collection	184
7	#6	Web of Science Core Collection	184

The results are limited to English-language publications. The reference lists of relevant articles and existing reviews were manually screened for additional guidelines.

The inclusion criteria were as follows: (1) explicit clinical practice guidelines or consensus statements on rectal cancer; (2) publication between 2010 and 2025; (3) generated by recognized national or international professional organizations or health agencies; and (4) addressing aspects such as definitions, staging, surgical approaches, adjuvant therapies, surveillance protocols, documentation, and palliative care.

The exclusion criteria were as follows: (1) studies that evaluated outcomes other than those of interest (surgical anatomy, adjuvant therapy, follow-up and surveillance, documentation, palliative care), (2) non-clinical studies, (3) in vitro studies, and (4) studies that were not published in Q1 or Q2 indexed journals. 

The findings of this review are presented in a narrative format and will examine the following areas of interest: surgical anatomy, adjuvant therapy, follow-up and surveillance, specific considerations, documentation, and palliative care.

For the present review, the following softwares were utilized: Microsoft Excel (Microsoft Corporation, Redmond, Washington, United States), Paperpile (Paperpile LLC, Cambridge, Massachusetts, United States), and jamovi Version 2.6.24 for Mac (The jamovi project (2025), Sydney, Australia (https://www.jamovi.org)). Artificial intelligence softwares were used for the conceptualization, investigation, review, and editing of the manuscript [3]. 

Results

Screening Process and Selection of Studies

**Figure 1:**
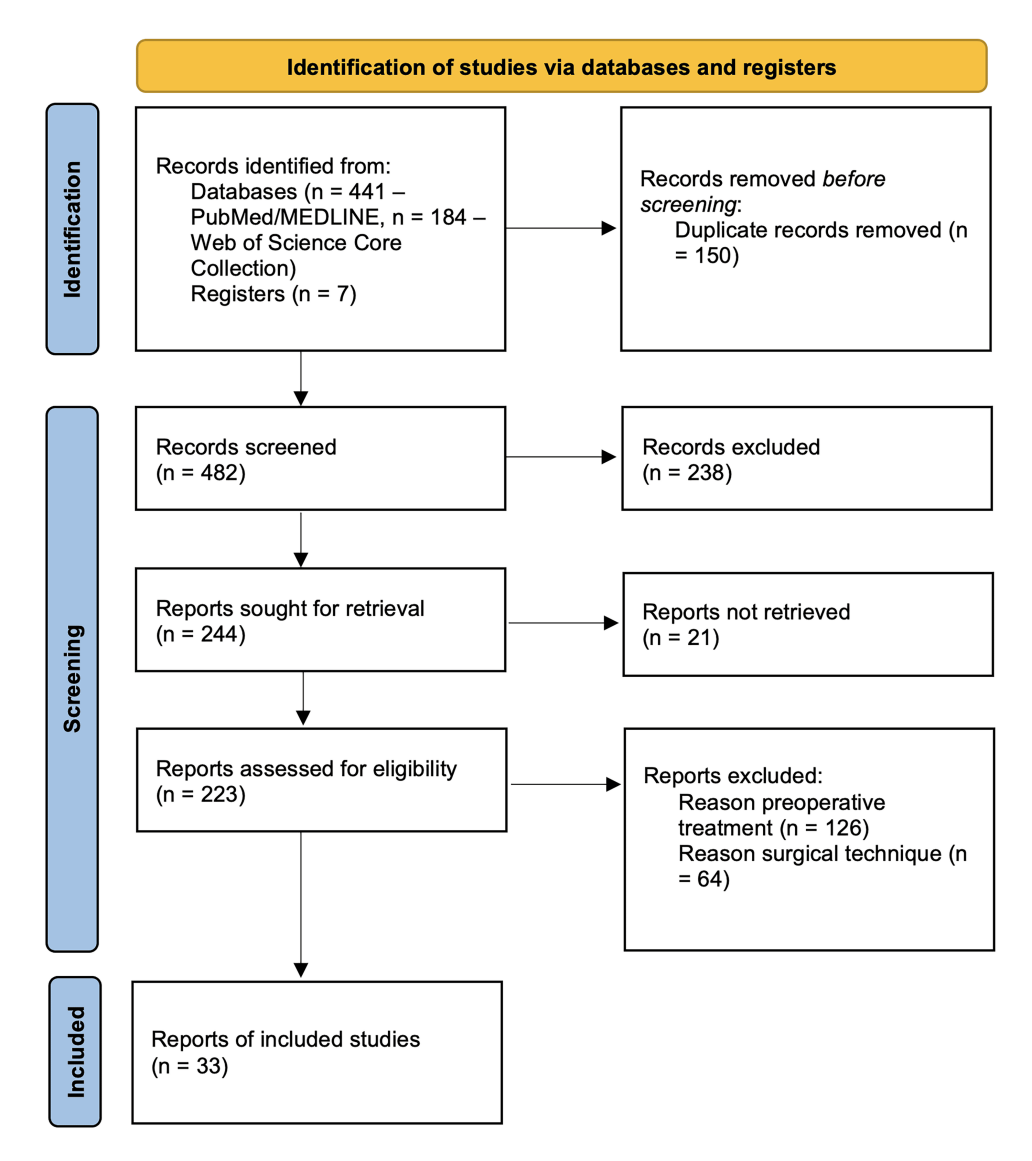
PRISMA 2020 flow diagram for the present review. PRISMA: Preferred Reporting Items for Systematic Reviews and Meta-Analyses

We retrieved 441 titles and abstracts from the PubMed/MEDLINE database, 184 from the Web of Science Core Collection, and seven from the Registers. Following the removal of 150 duplicate records, 482 titles and abstracts were screened. A total of 238 records were excluded because they did not address rectal cancer management or the outcomes of interest. Nineteen papers were not retrieved, and the full text of the remaining 225 was evaluated. Of these, 126 papers were categorized under preoperative treatment, and 64 scientific articles were categorized under surgical techniques. Ultimately, 35 studies were included in the qualitative analysis. Figure 1 shows the Preferred Reporting Items for Systematic Reviews and Meta-Analyses (PRISMA) 2020 flow diagram for the present review [4].

Surgical Anatomy of the Rectum

**Figure 2:**
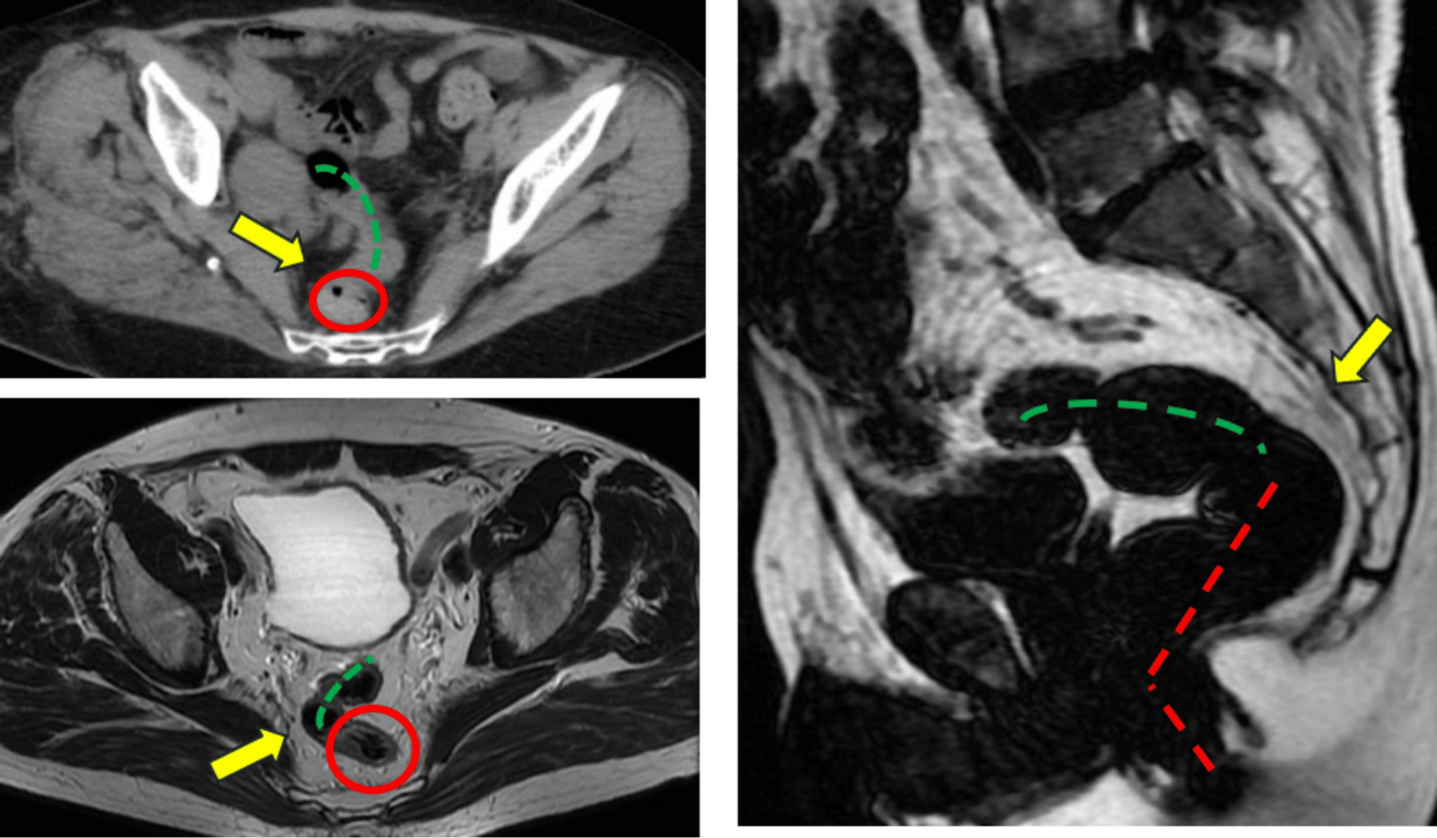
CT and MRI axial view and MRI sagittal view: sigmoid take-off, yellow arrow; rectum, red circle/dotted line; and sigmoid colon, green dotted line. Images are from the authors' patients and are not published elsewhere.

Despite significant heterogeneity among studies regarding the precise definition and reporting of rectal tumors as well as substantial variability in clinical practice concerning anatomical boundaries, a growing consensus has been reached in recent years. Increasingly, the upper boundary of the rectum is standardized and clinically recognized as the point at which the sigmoid colon commences, commonly termed the "sigmoid take-off" (see Figure 2) [5]. CT and MRI revealed the sigmoid takeoff as a visible anatomical marker. This marks the point at which the mesorectum transforms into the mesocolon. On sagittal images, the sigmoid take-off is identified as the area where the sigmoid colon makes a horizontal anterior transition. In the axial view, it is represented as a ventral projection [6]. The sigmoid take-off, as characterized by MRI, was validated by specimen analysis and is an anatomical landmark that can differentiate between rectal and sigmoid tumors to guide treatment and research [7].

The upper limit of the rectum is typically considered to be 15 cm from the anal verge. A tumor is classified as rectal cancer when its lower border is situated within 15 cm of the anal margin [8]. The surgical anal canal is defined as the region extending from the anal verge to the anorectal junction or ring. This anatomical landmark can be identified through digital rectal examination or MRI and is recognized as the superior boundary of the anal sphincter and puborectalis muscle. The surgical anal canal typically measures 4 cm in length.

The lower rectum is anatomically defined as the segment extending proximally from the anal verge for approximately 5 cm along the posterior rectal wall. Conversely, the UK Low Rectal Cancer (LOREC) initiative advocates the utilization of MRI landmarks for a more precise delineation of the lower rectum. Specifically, it recommends identifying the region where the mesorectum narrows at the commencement of the levator ani muscles adjacent to the pelvic sidewalls, visible on both the coronal and sagittal MRI planes. Typically, this anatomical landmark is positioned within approximately 6 cm from the anal margin [8-11]. 

The middle rectum is anatomically defined as the segment extending between 5 cm and 10 cm proximal to the anal margin, measured along the posterior rectal wall. The upper rectum encompasses the region situated between 10 and 15 cm proximal to the anal margin and serves as an anatomical transition zone connecting the rectum to the sigmoid colon.

Rigid proctoscopy remains the preferred method for accurately determining the tumor distance from the anal verge. To ensure consistency and reproducibility in reporting, an explicit documentation of the measurement technique is essential. Two independent predictive factors for a positive circumferential resection margin include tumor proximity to the anal verge and its circumferential location within the rectum, especially when positioned anteriorly between the 10 and two o'clock orientation. Therefore, these factors should be systematically documented before initiating therapeutic interventions [12].

The rectum, together with its associated lymphatic and vascular structures, is enveloped by the mesorectal fascia. The surgical technique known as total mesorectal excision (TME) involves the en bloc removal of the rectum and its surrounding mesorectal tissue, meticulously preserving the integrity of the mesorectal fascia. 

The rectum is categorized into intraperitoneal, partially covered, and extraperitoneal segments based on its anatomical relationship with the peritoneal reflection. Peritoneal reflection may serve as an imagistic landmark, anteriorly present at the level of the middle rectum, while lateral and posterior reflections are present at the level of the upper rectum. 

Anal verge represents the distal limit of the rectum. Surgical management of low rectal tumors is profoundly influenced by the anatomical and functional characteristics of the anal sphincter complex, which comprises internal and external sphincters, because of their crucial role in maintaining fecal continence.

Lateral pelvic lymph nodes, particularly those along the internal iliac and obturator regions, play an essential role in the staging and surgical planning for rectal cancer, especially in advanced low rectal tumors. In cases classified as N-positive (N+), regional lymphatic involvement encompasses several distinct nodal stations, including mesorectal lymph nodes, nodes located along the superior rectal vessels within the sigmoid mesocolon, and lymph nodes in the obturator, internal iliac, and inguinal areas. Specifically, inguinal lymph nodes are considered regional for tumors extending distally beyond the dentate line. Conversely, involvement of the external iliac, common iliac, or inguinal nodes (in tumors proximal to the dentate line) is categorized as distant metastatic lymphadenopathy (M1), reflecting nonregional nodal dissemination [12]. However, there is continuous debate between Eastern and Western centers regarding the benefits and indications of lateral pelvic lymph node dissection, and novel adjunctive techniques, such as indocyanine green fluorescence guidance, may improve the number of resected lymph nodes while diminishing the associated morbidity [13].

Adjuvant Therapy

For patients with high-risk stage II and III rectal cancers, adjuvant chemotherapy is recommended and should be integrated with prior preoperative treatment protocols. However, the efficacy of adjuvant chemotherapy in enhancing survival outcomes in this patient group has shown variable results across different studies. Some investigations have explored the role of postoperative chemotherapy, even within the context of total neoadjuvant therapy (TNT). Numerous ongoing trials are investigating organ preservation and watch-and-wait strategies in rectal cancer, as well as appropriate treatment approaches for cases of tumor regrowth.

Current evidence indicates substantial survival benefits associated with adjuvant chemotherapy for patients with both high-risk stage II and III rectal cancers, underscoring its potential value in comprehensive treatment strategies [14-16]. 

Nevertheless, evidence from several studies suggests that the efficacy of adjuvant chemotherapy is influenced by distinct tumor-specific risk factors. Specifically, in patients diagnosed with stage III rectal cancer, adjuvant chemotherapy has demonstrated a significant improvement in overall survival, particularly among those categorized as intermediate- and high-risk [17,18]. The utilization of adjuvant chemotherapy has progressively increased in recent years; however, substantial variability persists across different hospitals and geographic regions [16].

Table 3 lists the studies investigating the effects of adjuvant chemotherapy in patients with rectal cancer.

**Table 3:** Studies investigating the effects of adjuvant chemotherapy in patients with rectal cancer. DSS: disease-specific survival; CRT: chemoradiotherapy; DFS: disease-free survival; OS: overall survival; yp: pathological classification after neoadjuvant therapy; TME: total mesorectal excision; FL or 5-FU: fluorouracil; FOLFOX: folinic acid, fluorouracil, and oxaliplatin; HR: hazard ratio; RT: radiotherapy

First author, year of publication	Included patients	Results	Conclusions
Tamburini, 2022 [19]	Stage III rectal cancer, with neoadjuvant CRT followed by surgical resection	No DFS and OS differences between chemotherapy (single drug or polychemotherapy, including schemes with oxaliplatin) and control groups	For radical proctectomy after neoadjuvant CRT, the addition of adjuvant chemotherapy fails to improve OS and DFS. Adjuvant chemotherapy shows no advantage for the general population or high-risk individuals
Manzini, 2020 [20]	Any T, any N, M0 rectal cancers, including high-risk and low-risk tumors	OS: adjuvant chemotherapy, 17% reduction in mortality risk (HR=0.83; 95% CI: 0.76 to 0.91; p<0.05). DFS: adjuvant chemotherapy, 25% reduction in disease recurrence (HR=0.75; 95% CI: 0.68 to 0.83; p<0.05). Meta-analysis excluding specific studies: HR for survival benefit=0.87 (95% CI: 0.7 to -0.96; p<0.05); HR after excluding all studies with statistically significant results=0.90 (95% CI: 0.83 to 0.97; p<0.05)	The analysis revealed significant methodological issues in key studies supporting adjuvant chemotherapy for rectal cancer. After sensitivity analysis, excluding these studies from the analysis did not alter the conclusion that adjuvant chemotherapy provides a survival benefit
Hong, 2019 [21]	ypStage II or III rectal cancer, receiving neoadjuvant CRT followed by TME	DFS: FL arm: 56.8%; FOLFOX arm: 68.2% (HR=0.63; 95% CI: 0.43 to 0.93; p=0.018). OS: FL arm: 76.4%; FOLFOX arm: 78.1% (HR=0.73; 95% CI: 0.45 to 1.19; p=0.21)	Adjuvant FOLFOX significantly improved six-year DFS compared to FL, in patients with preoperative CRT and TME. FOLFOX was particularly beneficial for patients with ypStage III disease. OS was not significantly different between FOLFOX and FL; FOLFOX showed favorable outcomes in certain subgroups
Ahn, 2017 [17]	Stage II and stage III rectal cancers	OS: with adjuvant chemotherapy=72.6 months; without adjuvant chemotherapy=36.4 months (p=0.0003)	Adjuvant chemotherapy was associated with a significantly longer OS in patients with stage II and stage III rectal cancer
Sun, 2017 [22]	Pathologic stage II and III rectal cancers, receiving neoadjuvant CRT followed by surgery	OS: adjuvant chemotherapy: 60% at 7 years; no adjuvant chemotherapy: 55% at 7 years (p<0.001); HR for adjuvant chemotherapy: 0.81 (95% CI: 0.72 to 0.91; p<0.001). Stage II disease: adjuvant chemotherapy: 68% at 7 years; no adjuvant chemotherapy: 58% at 7 years (p<0.001); HR for adjuvant chemotherapy: 0.70 (95% CI: 0.57 to 0.87; p=0.002). Stage III disease: adjuvant chemotherapy: 56% at 7 years; no adjuvant chemotherapy: 51% at 7 years (p=0.017); HR for adjuvant chemotherapy: 0.85 (95% CI: 0.74 to 0.98; p=0.026)	Adjuvant chemotherapy improved OS in patients who received neoadjuvant CRT and surgery. At 7 years, patients receiving adjuvant chemotherapy had better survival compared to those who did not (60% versus 55%). The survival benefit was significant for stage II and stage III patients, with a more pronounced effect in stage II patients
Wu, 2017 [23]	Stage II-IV rectal cancers: 36.1%, stage II; 50.2%, stage III; and 13.7%, stage IV	Stage III and stage IV: adjuvant RT+chemotherapy=DSS than neoadjuvant CRT (p=0.03). Stage II: adjuvant RT+chemotherapy versus neoadjuvant CRT=similar DSS. Stage IIIA: adjuvant RT+chemotherapy=improved DSS with 3.2% and OS with 5.6% when compared with neoadjuvant CRT (p=0.04); adjuvant RT+chemotherapy=improved DSS with 18.9% and OS with 30% when compared with surgical resection alone. Stage IIA: adjuvant RT+chemotherapy versus neoadjuvant CRT=similar DSS; both improved DSS with 7% compared to surgery alone. High-risk patients (T3N+/T4): neoadjuvant CRT: better DSS than adjuvant RT+chemotherapy; surgery only: worst DSS for all groups	Neoadjuvant CRT usually provides better DSS than adjuvant RT+chemotherapy, especially in stages III and IV. Stage IIIA tumors benefit more from adjuvant RT+chemotherapy compared to neoadjuvant CRT. High-risk patients (T3N+/T4) have better DSS with neoadjuvant CRT compared to adjuvant CRT+chemotherapy
Hajibandeh, 2015 [14]	Stage II and stage III disease	Stage II DFS: OR=0.51 (95% CI: 0.39 to 0.67; p<0.001). Stage II OS: OR=0.64 (95% CI: 0.51 to 0.80; p<0.001). Stage III DFS: OR=0.61 (95% CI: 0.51 to 0.73; p<0.001). Stage III OS: OR=0.76 (95% CI: 0.61 to 0.96; p<0.02)	Adjuvant chemotherapy significantly improves DFS and OS in both stage II and stage III patients compared to surgery alone
Breugom, 2015 [24]	Histologically proven stage II or III rectal adenocarcinoma	OS: observation: 79.2%; chemotherapy: 80.4% (HR=0.93; 95% CI: 0.62 to 1.39; p=0.73). DFS: HR: 0.80 (95% CI: 0.60 to 1.07; p=0.13). Locoregional recurrences: observation: 7.8%; chemotherapy: 7.8%. Distant recurrences: observation: 38.5%; chemotherapy: 34.7% (p=0.39)	PROCTOR-SCRIPT trial found no significant benefit of adjuvant chemotherapy on OS, DFS, or recurrence rates compared to observation in rectal cancer patients. Five-year OS rates were similar in the observation group (79.2%) and the chemotherapy group (80.4%). The trial did not complete planned accrual, which may have affected its findings
Tiselius, 2013 [16]	Stage III rectal cancer patients	5-year OS: adjuvant chemotherapy: 65.8% (95% CI: 50% to 84%); no chemotherapy: 45.6% (95% CI: 39% to 52%). HR for death: adjuvant chemotherapy: 0.65 (95% CI: 0.5 to 0.8)	Adjuvant chemotherapy significantly increased use in stage III rectal cancer, with variations between regions. Adjuvant chemotherapy had a higher 5-year OS rate (65.8%) compared to no adjuvant therapy (45.6%). The risk of death was reduced with 35% in patients treated with adjuvant chemotherapy, as indicated by a HR of 0.65
Sauer, 2012 [25]	Stage II and stage III rectal cancer	10-year OS: preoperative CRT: 59.6%; postoperative CRT: 59.9% (p=0.85). 10-year cumulative incidence of local relapse: preoperative CRT: 7.1%; postoperative CRT: 10.1% (p=0.048). 10-year cumulative incidence of distant metastases: preoperative CRT: 29.8%; postoperative CRT: 29.6% (p=0.9). DFS: no significant differences	Preoperative CRT significantly improved local control compared to postoperative CRT, with a lower 10-year cumulative incidence of local relapse. There was no significant difference in 10-year OS between preoperative and postoperative CRT. There were no significant differences in the 10-year cumulative incidence of distant metastases or DFS between the groups
Petersen, 2012 [26]	Non-metastatic rectal carcinoma	OS: adjuvant versus no chemotherapy: 17% reduction in risk of death (HR=0.83; 95% CI; 0.76 to 0.91). DFS: adjuvant chemotherapy: 25% reduction in risk of disease recurrence (HR=0.75; 95% CI: 0.68 to 0.83)	Postoperative chemotherapy significantly reduced the risk of death by 17%. Adjuvant chemotherapy reduced the risk of disease recurrence by 25%. The study supports the use of 5-FU-based postoperative adjuvant chemotherapy for non-metastatic rectal carcinoma

In patients diagnosed with stage I rectal cancer, adjuvant therapy is typically not indicated after adequate surgical resection.

However, postoperative radiotherapy should be considered in stage II or III rectal cancers exhibiting high-risk features such as positive circumferential resection margins, tumor perforation, incomplete mesorectal excision, extranodal tumor deposits, or extranodal extension approaching the mesorectal fascia. Additionally, radiotherapy is recommended in cases with a heightened risk of local recurrence, particularly when neoadjuvant treatment was not administered [27,28].

Follow-Up and Surveillance

All patients treated for rectal cancer should undergo rigorous postoperative surveillance to detect recurrence, particularly within the initial five-year period following treatment [29].

For patients categorized as medium-risk, postoperative surveillance protocols should minimally incorporate the following measures [28]: (i) clinical evaluations, including digital rectal examinations and carcinoembryonic antigen (CEA) monitoring, performed biannually for the initial two years and annually thereafter for an additional three years; (ii) CT scans of the thorax, abdomen, and pelvis conducted semiannually for the first two years and annually for the subsequent three years [30]; (iii) pelvic MRI performed semiannually within the initial two-year interval, followed by annual scans for three additional years, specifically in patients at elevated risk for local recurrence, such as those with positive circumferential resection margins; and (iv) colonoscopy, mandatory within six months postoperatively if not completed preoperatively, subsequently repeated annually for the first three years, and every five years thereafter, with complete resection of any identified polyps [30]. For patients classified as high-risk, these surveillance intervals may be appropriately reduced by half [31]. Additionally, patients who select a nonoperative management approach require supplementary and tailored surveillance measures beyond the general protocols detailed above.

Routine assessment of quality of life using validated instruments, such as the European Organization for Research and Treatment of Cancer (EORTC) QLQ-C30 and QLQ-CR29 questionnaires, is strongly recommended to systematically evaluate the physical, emotional, and social dimensions of well-being in rectal cancer survivors.

Furthermore, patients who undergo low anterior resection should be routinely evaluated for low anterior resection syndrome (LARS) using the validated LARS score, specifically designed to quantify postoperative bowel dysfunction. Initial management strategies for LARS may incorporate symptomatic interventions, including dietary modifications, antispasmodic medications, antidiarrheal agents, or laxatives tailored according to individual patient needs [32].

Specific Considerations

For stage IV (M1) rectal cancers, comprehensive evaluation by an MDT, including surgeons with specific expertise in managing metastatic disease sites (e.g., hepatic surgery), is strongly recommended. Prior to initiating treatment, MDT should clearly define whether the therapeutic intent is potentially curative or palliative. An individualized therapeutic strategy that addresses all involved disease sites should be formulated accordingly [31]. 

Patients with rectal tumors accompanied by unresectable metastases typically require palliative management. In asymptomatic cases, therapeutic approaches that do not include surgical resection of either the primary or metastatic lesions should be considered; however, the potential outcomes of each therapeutic option must be thoroughly discussed with patients to facilitate shared decision-making. Notably, the resection of an asymptomatic primary rectal tumor carries a 5% risk of severe postoperative complications, potentially delaying the initiation of systemic therapy. Conversely, approximately 20% of patients who forego initial surgical resection of asymptomatic primary tumors eventually develop symptoms such as obstruction, perforation, hemorrhage, or pain, necessitating subsequent surgical intervention [32]. Surgical resection of the primary lesion combined with nonsurgical management of metastatic sites is indicated for patients experiencing significant rectal symptoms refractory to conservative therapies (e.g., severe bleeding, perforation, tumor penetration, or substantial luminal stenosis) [31]. Finally, in asymptomatic patients presenting with unresectable metastatic disease, systemic chemotherapy typically constitutes the initial therapeutic intervention.

Patients diagnosed with rectal cancer presenting with acute tumor-related complications should undergo optimal oncological management whenever feasible. In the setting of acute complicated rectal neoplasms where cancer is strongly suspected but lacks histopathological confirmation and conservative measures have proven inadequate, exploratory laparotomy should be considered, provided the patient is physiologically fit for surgery [33]. Life-threatening emergencies must receive immediate attention to ensure the simultaneous preservation of opportunities for multimodal oncological treatments. However, emergency resection performed for locally advanced rectal cancer may yield inferior oncological outcomes. 

Bleeding episodes are primarily managed with radiation therapy, achieving effective hemorrhage control in 87-100% of cases [34,35], with endoscopic intervention or angiography representing viable alternative strategies. Emergency surgical resection is typically unnecessary in such cases [27].

A tailored surgical approach should be adopted for obstructive rectal tumors. This may involve one-stage resection with primary anastomosis, potentially accompanied by a proximal diversion stoma; a Hartmann procedure; two-stage resections following an initial decompressing colostomy; or limited resection subsequent to successful stent placement [33]. For obstructing tumors located in the middle or lower rectum, a proximal diverting stoma should be considered to achieve effective decompression [27]. In patients presenting with unresectable tumors or those with significant operative risk, alternative approaches, such as a double-barrel stoma or endoscopic stenting, may provide suitable palliation.

In cases involving tumor perforation, immediate rectal resection, with or without primary anastomosis, is mandated. Diastatic perforations typically require extensive resection. 

Comprehensive multivisceral pelvic resections may be required for patients with locally advanced or recurrent disease; however, these extensive surgical interventions should be pursued only within specialized multidisciplinary centers, where complete (R0) resection is anticipated and appropriate expertise and resources are available [31,32].

Documentation

The operating surgeon may document in the operative report specific parameters recommended by the Quality Assessment and Safety Committee of the American Society of Colon and Rectal Surgeons, including the patient's American Society of Anesthesiologists (ASA) score; nature of surgery (elective or emergency, specifying indications such as obstruction, bleeding, or perforation); type of surgical procedure performed (low anterior resection, abdominoperineal resection, and local excision, among others); operative approach utilized (open, laparoscopic, robotic, transanal); anatomical tumor location within the rectum (upper, middle, or lower segment); precise measurement of the tumor's inferior margin distance from the anal verge (0-15 cm); mobilization status of the splenic flexure (yes/no); level of transection of the inferior mesenteric artery (high/low ligation); distance of rectal transection distal to the tumor margin (in centimeters); reconstruction technique employed (stapled or handsewn anastomosis, side-to-end, end-to-end, colonic J-pouch); presence and type of diversion stoma created (ileostomy or colostomy); details of any multivisceral resections performed (e.g., urinary bladder, small bowel, vagina, prostate); metastasectomy procedures conducted (hepatic, peritoneal, etc.); completeness of tumor resection (R0, R1, or R2 margins); occurrence and nature of intraoperative complications (e.g., ureteric injury, small bowel injury, vascular injury, inadvertent tumor or rectal perforation); requirement for intraoperative blood transfusions; photographic documentation of the TME specimen (yes/no); and additional relevant details provided in a free-text narrative format [36].

The pathology report may include the following [37]: (i) Macroscopic assessment may include specification of the surgical procedure performed and identification of resected specimens; exact tumor location; tumor dimensions; distance between the tumor and nearest longitudinal margin; occurrence of intraoperative specimen perforation; tumor position relative to the peritoneal reflection; evaluation of adherence to proper dissection plane (mesorectal, intramesorectal, or muscularis propria); and measurement of the distance between the inferior tumor margin and the dentate line following abdominoperineal resection (APR). (ii) Microscopic evaluation may detail the histological type of tumor, grade of differentiation, pathological T stage, tumor response to neoadjuvant therapy, involvement of longitudinal and circumferential resection margins, lymph node status including total number dissected, number invaded, and status of the apical lymph node, presence of venous invasion, and results from additional biopsies of suspected metastatic sites. Pathological reporting can integrate the standardized elements recommended by the Reporting Proforma for Colorectal Carcinoma Resection Specimens provided by the Royal College of Pathologists [37,38].

Palliative Care

Palliative surgical resection should generally be avoided unless necessary to alleviate specific tumor-related symptoms such as obstruction or significant hemorrhage. Endoscopic stenting is a viable palliative alternative for patients presenting with inoperable obstructive rectal tumors. For advanced rectal cancer patients in whom curative resection is unattainable, optimal supportive care, including systemic chemotherapy and targeted symptom management, is recommended to enhance quality of life and manage disease-related complications effectively.

Health System and Implementation

The establishment of a national rectal cancer database is recommended to systematically monitor patient outcomes, uphold and enhance the quality of clinical care, and support both research initiatives and informed healthcare policy making. Surgeons and healthcare institutions should ensure meticulous and accurate electronic data collection to enable centralized data transfer, comprehensive analysis, and subsequent improvements in clinical practice [39]. 

Surgeons engaged in rectal cancer surgery should complete specialized training programs and obtain certification specifically related to rectal cancer management, encompassing TME and minimally invasive surgical techniques, thereby ensuring proficiency and high standards of care. 

Institutions offering surgical treatment for rectal cancer should adhere to clearly defined accreditation criteria, including the availability of MDT, advanced imaging modalities, and proficiency in contemporary surgical approaches. 

Regular institutional audits assessing surgical specimens, specifically focusing on mesorectal excision quality grading and circumferential resection margin evaluation, are mandatory to systematically monitor performance, enhance surgical outcomes, and maintain quality standards.

Discussion

Benefits of Using Standardized Approaches in Rectal Cancer Management

Implementing standardized, guideline-based strategies in the management of rectal cancer has been shown to significantly enhance patient outcomes. Research has consistently indicated that adherence to established treatment guidelines is associated with improved survival rates [40]. For example, a population-based study conducted in California revealed that patients receiving therapy in accordance with the National Comprehensive Cancer Network (NCCN) guidelines exhibited significantly better disease-specific survival compared to those treated outside of these guidelines. Conversely, deviations from established guidelines often result in poorer outcomes, a disparity frequently observed in underserved populations [40]. Furthermore, the guidelines underscore the importance of an MDT approach, which involves collaboration among surgeons, radiologists, pathologists, and oncologists to develop consensus treatment plans. The MDT model itself enhances the quality of care, with pooled analyses demonstrating that routine tumor board discussions lead to more accurate staging, increased guideline adherence, and management changes that improve outcomes in a substantial subset of patients [41]. 

Perhaps the clearest example of a standardized approach benefiting patients is the universal adoption of TME as a surgical cornerstone. The meticulous TME technique, now a global standard, dramatically reduced local recurrence rates from 20-30% to <5% in modern series ​[42]. This improvement, achieved by sharp dissection along embryologic planes with intact mesorectal fascia, underscores how establishing technical surgical standards can directly translate into better oncological outcomes. In populations where rectal cancer surgery was centralized to high-volume centers with quality control audits, local recurrence and positive margin rates dropped significantly, while survival improved​ [43]. For example, region-wide centralization in Spain that concentrated rectal surgery in experienced units increased complete TME specimen rates from 36.2% to 85.7% and significantly reduced unplanned surgeries​ [43]. Along with improved local control, the standardized use of neoadjuvant chemoradiation for stage II-III disease (based on German and other trial data) has halved local recurrence relative to postoperative treatment ​[44], and standardized criteria for adjuvant chemotherapy have yielded clear survival improvements. Notably, large analyses have confirmed that adjuvant chemotherapy after TME and chemoradiation improves overall survival, even in patients with an excellent pathological response. Taken together, these findings highlight that a systematic, evidence-based approach that encompasses uniform definitions, treatment algorithms, and quality metrics reduces unwarranted variations in care and improves clinical outcomes across diverse practice settings​ [45]. In support of this, multiple professional bodies around the world have published concordant rectal cancer guidelines in the past decade, reflecting an international consensus on best practices. By adhering to such standardized protocols, clinicians can ensure that each patient receives optimally coordinated therapy, thereby maximizing the curative potential while minimizing avoidable morbidity.

Role of Education in Shaping Future Rectal Cancer Surgeons

Ongoing surgical education and specialized training are pivotal for translating the guideline principles into high-quality surgical care. The technical complexity of rectal cancer surgery, which often requires pelvic dissection in a narrow field and multidisciplinary management, means that the surgeon's experience and training directly impact patient outcomes. High-volume rectal surgeons consistently achieved superior results, including lower perioperative mortality (OR: 0.43; 95% CI: 0.21-0.87) and higher rates of sphincter preservation (OR: 0.65; 95% CI: 0.48-0.89)​ [46]. This volume-outcome relationship underlies the push for formal subspecialty training in colorectal surgery. Fellowship-trained colorectal surgeons not only have additional technical proficiency in TME but are also versed in nuanced decision-making, such as managing borderline resectable tumors or tailoring multimodal therapy, which translates to better oncologic outcomes. Indeed, recent data confirm that patients treated by board-certified colorectal specialists have significantly improved survival rates compared to those treated by general surgeons [47]. 

Educational initiatives at the national level have further institutionalized quality. The American College of Surgeons' National Accreditation Program for Rectal Cancer (NAPRC) now mandates specific training and credentialing standards, including case volume minimums, MDT care pathways, and continuous outcome monitoring for centers seeking rectal cancer accreditation​ [48]. Early evidence from Europe showed that structured training programs could dramatically improve outcomes, and the UK Low Rectal Cancer Programme and Sweden's TME training workshops led to marked reductions in positive margins and local recurrence in previously low-performing hospitals ​[43]. These improvements persisted over time, illustrating how mentoring surgeons in optimal techniques and perioperative planning has a lasting impact on care quality [49,50]. Modern educational efforts also emphasize proficiency in minimally invasive approaches, such as laparoscopic, robotic, and transanal TME, and proper patient selection for emerging treatments. Simulation-based training, proctoring by expert surgeons, and specialized fellowships impart skills that shorten the learning curve for advanced techniques, ensuring that the next generation of rectal surgeons can safely adopt innovations without compromising oncological outcomes. Ultimately, fostering a culture of continuous education through formal training, case review conferences, and involvement in guideline development equips surgeons with the ability to deliver standardized, high-quality care. This investment in human capital synergizes with guidelines; well-trained surgeons are able to execute guideline-recommended therapy, and in turn, successful outcomes reinforce adherence to these standards [45]. 

Clinical Implications and Future Perspectives

The insights gained from this umbrella review have several important clinical implications. First, the clear benefits of guideline adherence and multidisciplinary coordination imply that greater efforts should be made to implement these standards uniformly. Health systems should invest in developing rectal cancer centers with excellence and referral networks so that patients have access to high-level multidisciplinary care. Adopting uniform terminology and standardized documentation (e.g., consistently reporting tumor height, mesorectal fascia involvement, and TME quality) will improve communication between clinicians and facilitate the comparability of outcomes across institutions. In practice, this means ensuring that all rectal cancer cases are presented to an MDT and managed according to the best-practice algorithms. For clinicians, staying abreast of evolving guidelines is crucial, particularly as new evidence rapidly emerges that can shift the standards of care. For instance, TNT has now entered guidelines as an acceptable approach for many locally advanced cases, and trials such as RAPIDO demonstrated that upfront short-course radiation plus chemotherapy lowered the risk of distant metastases and improved disease-related treatment failure rates at three years​ [51]. Similarly, the PRODIGE 23 trial showed a significant improvement in pathological complete response and three-year disease-free survival with TNT, presenting a paradigm shift where complete systemic and radiation therapy is delivered before surgery for maximal tumor downstaging ​[52]. Clinicians should anticipate that future updates of guidelines will increasingly endorse TNT for appropriate patients, and they should prepare to incorporate these protocols into practice.

Organ-preserving management is another evolving area with a major impact on the future. The possibility of nonoperative management ("watch-and-wait") in patients who achieve a complete clinical response to chemoradiation is one of the most significant recent developments. Although still somewhat controversial, growing evidence supports its safety in well-selected patients under strict surveillance. The OPRA trial (US phase II study) recently reported organ preservation in approximately half of the patients treated with TNT, with no compromise in five-year survival relative to standard surgery cohorts​ [53]. These findings suggest that a subset of rectal cancer patients, especially those with distal tumors where surgery risks a permanent stoma, might be managed nonoperatively to preserve rectal function and quality of life. The clinical implication is that treatment teams should develop protocols for response assessment, including high-quality MRI and endoscopy, and follow-up if pursuing watch-and-wait and patients should be counseled about the risks and benefits of this approach [54]. 

Advances in molecular oncology and technology have opened up new frontiers. The advent of effective immunotherapy for microsatellite-unstable (dMMR) rectal cancer is a prime example. A groundbreaking study reported a 100% clinical complete response rate in a small series of dMMR rectal cancer patients treated with checkpoint blockade alone, obviating the need for chemoradiation or surgery in these individuals [55]. Although longer follow-up and larger studies are needed, this suggests that, in the foreseeable future, a subset of rectal cancers defined by genetic biomarkers may be cured with nonsurgical modalities alone. Accordingly, MDT should incorporate routine MMR testing and consider referrals to immunotherapy trials or protocols for eligible patients. Beyond immunotherapy, the increasing use of circulating tumor DNA (ctDNA) assays may refine postoperative management by identifying minimal residual disease, allowing intensification or de-intensification of therapy on a personalized basis [2]. In terms of surgical innovation, minimally invasive techniques continue to improve recovery without sacrificing the outcomes. Laparoscopic and robotic TME are already widely adopted, and evidence shows that they can achieve oncologic results equivalent to open surgery in expert hands. The clinical message is that surgeons should be trained in these approaches so that patients can reap benefits, such as faster recovery and reduced wound complications. That said, vigilance is needed in introducing novel techniques; the learning curve must be respected, and a rigorous audit of oncological outcomes should accompany any new surgical approach.

The future of rectal cancer management is trending toward more personalized and less invasive therapy but is always anchored by the principles of multidisciplinary, standardized care. Ongoing research will likely expand the indications for selective nonoperative management and systemic therapies tailored to tumor biology. To translate these advances into practice safely, institutions will need to uphold the pillars discussed here: adherence to evolving evidence-based guidelines, investment in surgeon education and subspecialty training, and continuous quality monitoring via audits. By doing so, we not only improve current care but also create an adaptable framework ready to integrate future innovations, ultimately further improving outcomes and quality of life for patients with rectal cancer.

Limitations of Such an Initiative and Limitations of the Current Study

Despite its clear benefits, implementing standardized guidelines and protocols has several limitations. Resource disparities between healthcare settings pose significant barriers, limiting the uniform adoption of multidisciplinary approaches, advanced imaging, and specialist training programs. Smaller or rural centers may lack the infrastructure or volume necessary to achieve full guideline adherence, potentially perpetuating care variability and adversely affecting outcomes. Moreover, standardization initiatives inherently depend on clinician engagement and institutional commitment, which may vary substantially across regions and institutions. Continuous education and audits, while beneficial, require significant administrative support and financial investment, potentially straining institutional resources.

Regarding the limitations of the current umbrella review, there are inherent methodological constraints. By synthesizing the existing guidelines, our review inherits individual weaknesses, including possible biases, inconsistent evidence quality, and variations in recommendation strengths across sources. Furthermore, guidelines evolve rapidly, meaning that the recommendations summarized here might become outdated as new evidence emerges. Another limitation is the exclusion of non-English-language publications, potentially omitting relevant international perspectives. Finally, heterogeneity in guideline development processes across organizations, such as differing consensus methodologies, limits direct comparability and influences the strength of our conclusions and recommendations. Addressing these limitations in future research could enhance guideline applicability and global generalizability.

## Conclusions

This umbrella review underscores the key components integral to the management of rectal cancer, with particular emphasis on the necessity of multidisciplinary collaboration and the adoption of standardized terminology to optimize patient outcomes. Clearly delineated surgical landmarks, including the sigmoid take-off and mesorectal fascia, are essential for precise tumor staging and meticulous surgical intervention. Although adjuvant chemotherapy demonstrates significant survival advantages, particularly in high-risk stage II and III patient cohorts, therapeutic approaches must be individualized. Comprehensive postoperative surveillance incorporating clinical evaluation, biomarker monitoring, imaging modalities, and colonoscopic examination is imperative for the timely identification of recurrences and assessment of quality of life. Additionally, personalized, multidisciplinary strategies are essential for addressing metastatic disease and acute tumor-related complications, such as obstruction, hemorrhage, and perforation. Standardization of the operative and pathological documentation is fundamental for consistent quality assurance and data reliability. Structured palliative care frameworks are critical for maintaining patients' quality of life in advanced disease stages. Finally, implementation of national registries, specialized surgical training, accredited centers of excellence, and systematic auditing processes are essential measures to further enhance healthcare delivery and clinical outcomes.
